# Adenosine Signaling and the Energetic Costs of Induced Immunity

**DOI:** 10.1371/journal.pbio.1002136

**Published:** 2015-04-27

**Authors:** Brian P. Lazzaro

**Affiliations:** 1 Department of Entomology, Cornell University, Ithaca, New York, United States of America

## Abstract

Life history theory predicts that trait evolution should be constrained by competing physiological demands on an organism. Immune defense provides a classic example in which immune responses are presumed to be costly and therefore come at the expense of other traits related to fitness. One strategy for mitigating the costs of expensive traits is to render them inducible, such that the cost is paid only when the trait is utilized. In the current issue of *PLOS Biology*, Bajgar and colleagues elegantly demonstrate the energetic and life history cost of the immune response that *Drosophila melanogaster* larvae induce after infection by the parasitoid wasp *Leptopilina boulardi*. These authors show that infection-induced proliferation of defensive blood cells commands a diversion of dietary carbon away from somatic growth and development, with simple sugars instead being shunted to the hematopoetic organ for rapid conversion into the raw energy required for cell proliferation. This metabolic shift results in a 15% delay in the development of the infected larva and is mediated by adenosine signaling between the hematopoietic organ and the central metabolic control organ of the host fly. The adenosine signal thus allows *D*. *melanogaster* to rapidly marshal the energy needed for effective defense and to pay the cost of immunity only when infected.

## Abstract 2

Fruit flies infected by a parasitoid wasp use adenosine signaling to recruit energy away from tissue growth in order to support proliferation of defensive immune cells, thereby paying a deployment cost of inducible immunity. Read the Research Article.

While sitting around the campfire, evolutionary biologists may tell tales of the Darwinian Demon [1], a mythical being who is reproductively mature upon birth, lives forever, and has infinite offspring. In the morning, though, we know that no such creature can exist. All organisms must make compromises, and fitness is determined by striking the optimal balance among traits with competing demands. This is the central premise of life history theory: adaptations are costly, and increasing investment in one trait often forces decreased investment in others [2].

Phenotype plasticity provides a partial solution to this evolutionary conundrum. If traits can be called upon only when required, the costs can be mitigated during periods of disuse. There are many examples of plastic costly adaptations, including the defensive helmets grown by *Daphnia* species in response to the presence of predators or abiotic factors that signal risk of predation ([3,4] and references therein), the flamboyant plumage that male birds exhibit during breeding season [5], and the inducible immune systems of higher plants and animals [6,7].

The very inducibility of immune systems implicitly argues for their cost. If immune defense was cost-free, it would be constantly deployed for maximum protection against pathogenic infection. However, immune reactions have frequently been inferred to be energetically demanding (e.g., [8,9]) and carry the risk of autoimmune damage (e.g., [10,11]). It may therefore be evolutionarily adaptive to minimize immune activity in the absence of infection, to rapidly ramp up immunity in response to infection, and then to quickly shut down the immune response after the infection has been managed [12,13]. A paper by Bajgar et al. in the current issue of *PLOS Biology* [14] uses the *Drosophila*–*Leptopilina* host–pathogen system to quantitatively measure the energetic expense of induced up-regulation of immunity, demonstrating plastic metabolic reallocation toward immune cell proliferation at the expense of nutrient storage, growth, and development.

Parasitoid wasps such as *Leptopilina* species infect their insect larval hosts by laying an egg inside the host body cavity [15]. Unimpeded, the egg hatches into a wasp larva that feeds off and develops inside the still-living host. In the case of *Leptopilina boulardi* infecting *Drosophila melanogaster*, the infected host larva survives to enter pupation, but if the parasitization is successful, an adult wasp will ultimately emerge from the pupal case instead of an adult fruit fly. This clearly is against the interest of the *D*. *melanogaster* host, so the larval fly attempts to encapsulate the *L*. *boulardi* egg in a sheath of specialized blood cells called lamellocytes that collaborate with other cell types to suffocate the wasp egg, deprive it of nutrients, and kill it with oxidative free radicals [16]. The struggle between fly and wasp is of the highest stakes, with guaranteed death and complete loss of evolutionary fitness for the loser.

Natural *D*. *melanogaster* populations are rife with genetic variation for resistance to parasitoids, and laboratory selection for as few as five generations can increase host survivorship from 1%–5% to 40%–60% (e.g., [17,18]). This evolved resistance comes at a cost, though. Larvae from evolved resistant strains have decreased capacity to compete with their unselected progenitors under crowded or nutrient-poor conditions [17,18]. The general mechanism for enhanced resistance has been recurrently revealed to be an increase in the number of circulating blood cells (hemocytes). But why are the resistant larvae outcompeted by susceptible larvae? One compelling hypothesis is that the extra investment in blood cell proliferation comes at an energetic cost to development of other tissues, a cost which may be exacerbated by a decrease in feeding rate of the selected lines [19] and that leads to impaired development under nutrient-limiting conditions. The cost can be limited, however, by producing the defensive blood cells in large numbers only when the host is infected and has need of them. Effectively achieving this requires the capacity to rapidly signal cell proliferation and to recruit the energy required for hematopoiesis from other physiological processes.

Bajgar et al. [14] use a series of careful experiments to document energetic redistributions and costs associated with hemocyte proliferation and lamellocyte differentiation in *D*. *melanogaster* infected by *L*. *boulardi*. They find that parasitoid infection results in a 15% delay in host development, and that at least a fraction of this delay can be plausibly attributed to a metabolic reallocation that supports blood cell proliferation over somatic growth. Specifically, they find that dietary carbohydrates are shunted away from energetic storage and tissue development, and instead are routed to the lymph gland, where hemocytes are being produced and differentiated. The signal to activate this reallocation emanates from the lymph gland and developing hemocytes themselves in the form of secreted adenosine, which is then received and interpreted by the central metabolic control organ, the fat body. Bajgar et al. [14] are able to show in unprecedented quantitative precision, that secreted adenosine acts as a signal to rapidly trigger inducible immune defense, that this immune induction is costly at the level of individual tissues and the whole organism, and that the cost of induced defense is paid only upon infection.

The *D*. *melanogaster* metabolic rearrangement after infection by *L*. *boulardi* is profound [14]. Within 6–18 hours of infection, host larvae show a strong reduction in the incorporation of dietary carbon into stored lipids and protein and an overall reduction in glycogen stores. The levels of circulating glucose and trehalose spike, with those saccharides seemingly directed to the hemocyte-producing lymph gland. Expression of glycolytic genes is suppressed in the fat body, while fat body expression of a trehalose transporter is up-regulated to promote trehalose secretion into circulation. Glucose and trehalose transporters are concomitantly up-regulated in the lymph gland and developing hemocytes to facilitate import of the circulating saccharides. Genes required for glycolysis—but not the TCA cycle—are simultaneously up-regulated in the lymph gland and nascent hemocytes to turn those sugars into quick energy under the Warburg effect [20]. The totality of the data is consistent with an interpretation that the induced hemocyte proliferation is directly costly to host larval growth as an immediate consequence of substantial energetic reallocation.

This same research group had previously shown that extracellular adenosine can be used by *Drosophila* as a signal in regulating inflammation-like responses [21]. In the present study, they use RNAi to knock down expression of the adenosine transporter *ENT2* specifically in the lymph gland and developing hemocytes. This prevention of adenosine export eliminates the spike in circulating glucose and trehalose after infection. Infected larvae with knocked down expression of *ENT2* continue to allocate dietary carbon to lipid and proteins as though they were uninfected, they fail to differentiate an adequate number of lamellocytes, and they suffer reduced resistance to the parasitoid. These data firmly implicate extracellular adenosine as a critical trigger mediating the switch in energetic allocation from growth and development to induced immunity. In further support of this interpretation, larvae that are mutant for the adenosine receptor *AdoR* show a nearly 70% reduction relative to wild type in the number of differentiated lamellocytes after infection and consequently encapsulate the parasitoid egg at nearly 4-fold lower rates. Like the *ENT2* knockdowns, *AdoR* mutant larvae fail to exhibit elevated levels of circulating glucose or trehalose when infected by *L*. *boulardi*, although surprisingly, the *AdoR* mutants do show an appropriate inhibition of glycogen storage when infected. Overall, the genetic results indicate that ENT2 protein allows secretion of adenosine from the lymph gland. This adenosine signal is received in the fat body, which then dampens energetic storage in favor of secretion and circulation of simple saccharides. These sugars are taken up by the lymph gland to rapidly facilitate hemocyte proliferation, lamellocyte differentiation, and immune defense against the parasitoid. In satisfying consistency with this model, Bajgar et al. [14] show that lamellocyte differentiation and host resistance can be partially rescued even in the absence of *ENT2* or *AdoR* by supplementing the *D*. *melanogaster* diet with extra glucose, thereby providing the boost in the circulating saccharides required for hematopoiesis.

There are key differences between studies of evolutionary tradeoffs, such as those conducted by Kraaijeveld and colleagues (e.g., [17–19]), and studies of physiological costs such as the one conducted by Bajgar et al. [14]. The evolutionary tradeoffs exposed by experimental selection are experienced even in the absence of parasitoid infection. Blood cell number is constitutively higher in the evolved resistant strains, which presumably allows a faster and more robust defense against a wasp egg. However, the resistant larvae always suffer the cost when reared in competitive conditions. Because the costs arise from a constitutive investment in defense, regardless of whether parasitoids are present, these are sometimes referred to as “maintenance” costs or fixed costs [22]. In contrast, Bajgar et al. have measured a physiological “deployment” cost [22], which is conditionally experienced only once the host activates an immune response.

It remains an open question the extent to which maintenance costs and deployment costs share mechanistic bases. In the present example, Bajgar et al. [14] show clearly that the machinery is in place to route energetic investment away from growth and development in favor of hemocyte proliferation. Extrapolating from the laboratory selection experiments [17–19], a natural increase in the epidemiological risk of parasitization in the wild might favor greater constitutive investment in hemocyte production. In such a scenario, it is easy to imagine the adaptive value of a mutation or genetic variant that results in higher expression of *ENT2* in the lymph gland in the absence of infection, driving constitutively higher hemocyte number and protection against infection at the expense of constitutively lower nutrient storage and growth. In this way, a plastic switch could be converted into a hardwired trait, a deployment cost would become a maintenance cost, and a physiological tradeoff would become an evolutionary one. However, it is important to appreciate that there typically are many solutions to any biological problem, and constitutively elevated blood cell number could also evolve via mechanisms unrelated to adenosine signaling. A key challenge for life history biologists will be to bridge physiological studies such as that by Bajgar and colleagues [14] with the evolutionary studies that preceded it, testing whether physiological and evolutionary tradeoffs share a mechanistic basis.

While in the bright light of day, evolutionary biologists can agree that the Darwinian Demon is just a ghost story, we have precious few examples of evolutionary or physiological tradeoffs where the mechanistic bases are well understood. More careful quantitative and genetic studies like that of Bajgar et al. [14] are necessary to carry us beyond reliance on abstract concepts like “resource pools” [2] and into mechanistic understanding of how life history tradeoffs operate on both the physiology of individuals and the evolution of species.
